# Supplementary material for: A spatial transcriptomic atlas of acute neonatal lung injury across development and disease severity
Source: bioRxiv. 2025 Jun 5:2025.06.02.656433. Preprint. [Version 1] doi: 10.1101/2025.06.02.656433 (PMC12157373; doi:10.1101/2025.06.02.656433)
Supplement: Supplement 1 [file media-1.pdf]

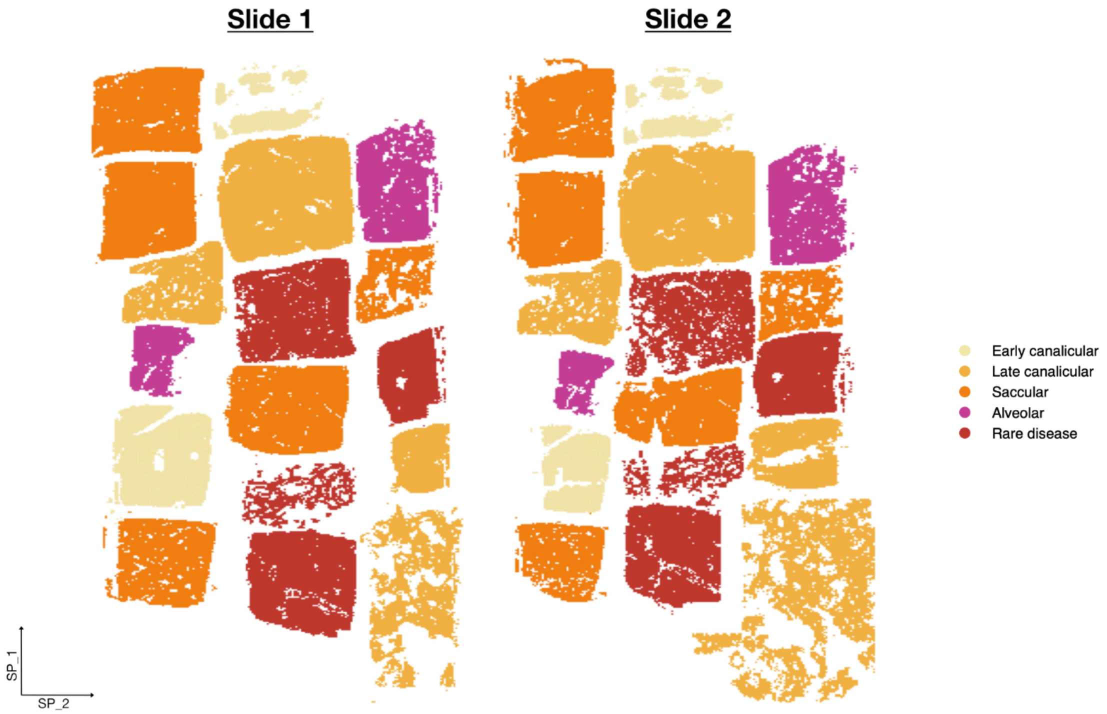

Supplemental Fig. S1:

A plot that shows each cell within each sample, highlighting the tissue structure of each sample across both slides. Each sample is colored by their gestational age and/or disease pathology.

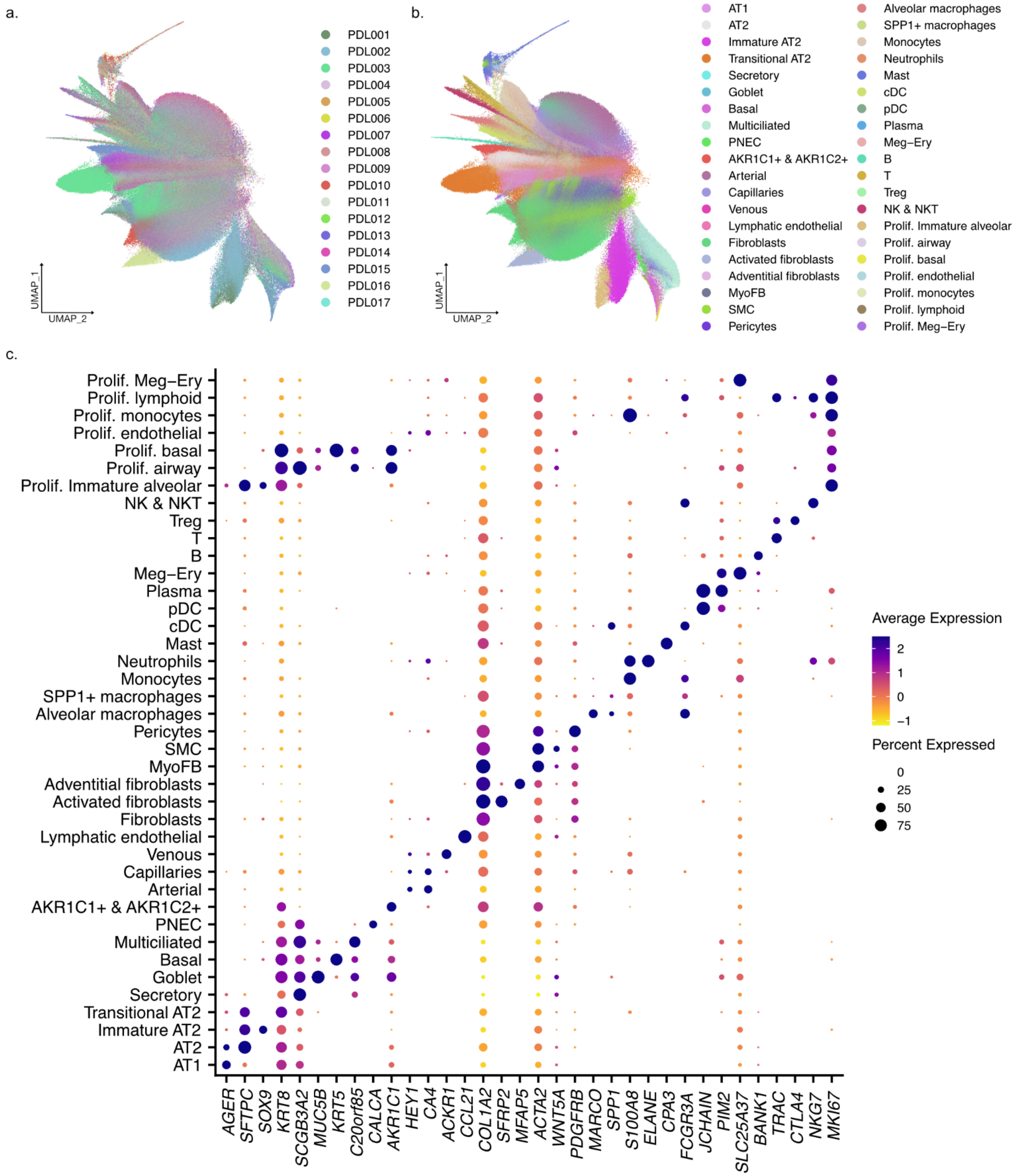

Supplemental Fig. S2:

a-b) UMAP plots showing the distribution of cells from each individual (a) and of each cell type (b). c) A dotplot highlighting hallmark genes used to annotate each cell type. Note - one cell type (*AKR1C1+ & AKR1C2+)* was labeled as such because of its high expression of AKR1C1, AKR1C2, and general airway epithelium markers (*EPCAM*, *SCGB3A2*, *SCGB1A1*, etc).

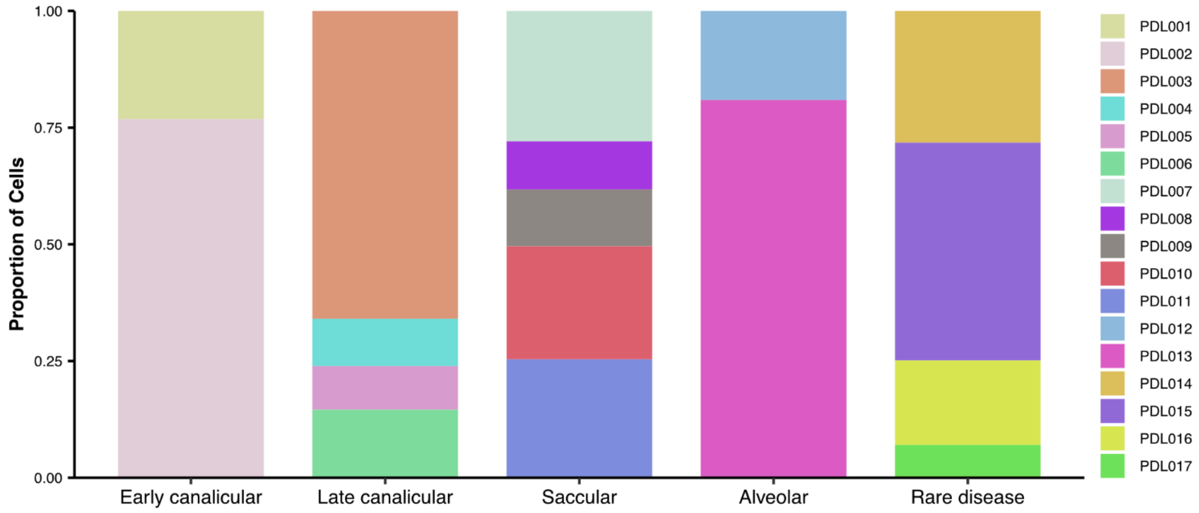

Supplemental Fig. S3:

A stacked bar chart showing the proportion of cells from each individual within each category.

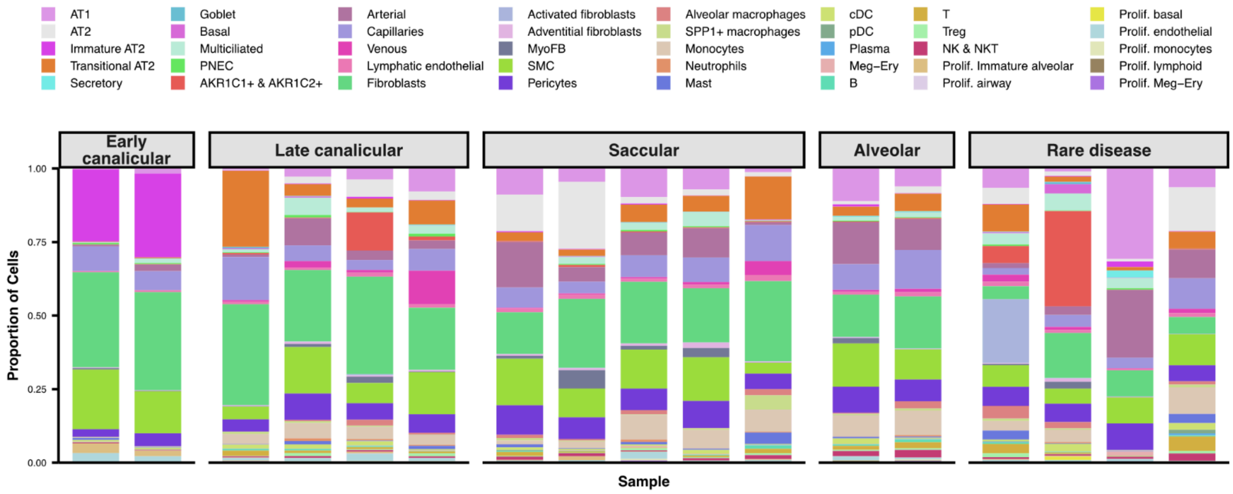

Supplemental Fig. S4:

A stacked barchart highlighting the proportion of each cell type across every sample in this cohort.

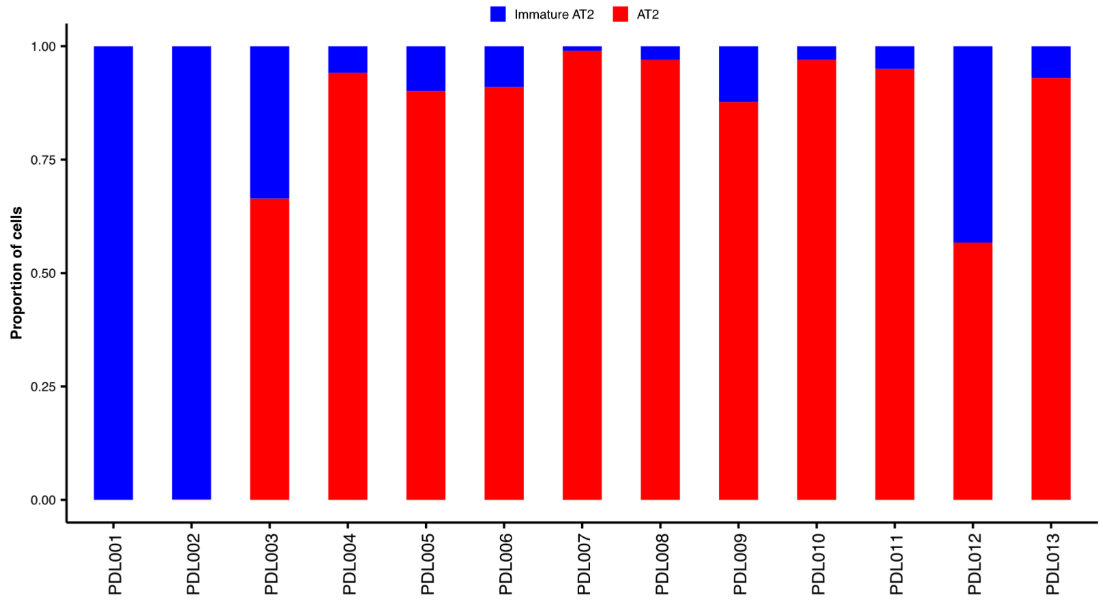

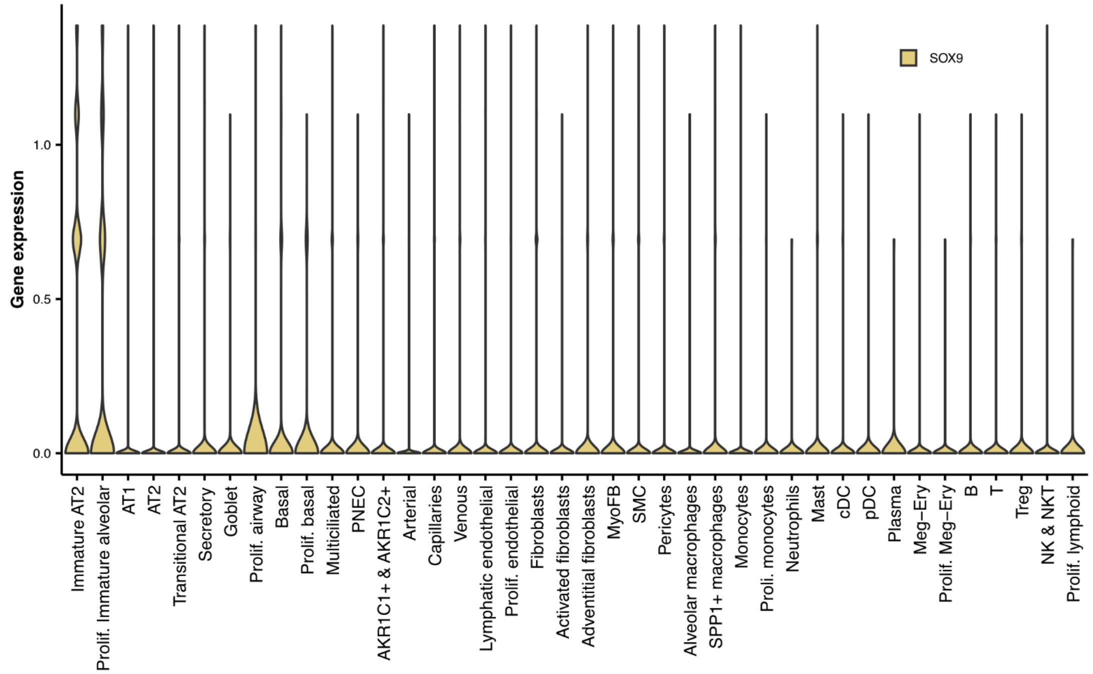

Supplemental Fig. S5:

a) A stacked bar chart showing the proportion of immature AT2 and AT2 cells across each individual, not including the rare diseased individuals. b) Violin plots showing the expression of *SOX9* across all cell types in cells from the same individuals used in (a).

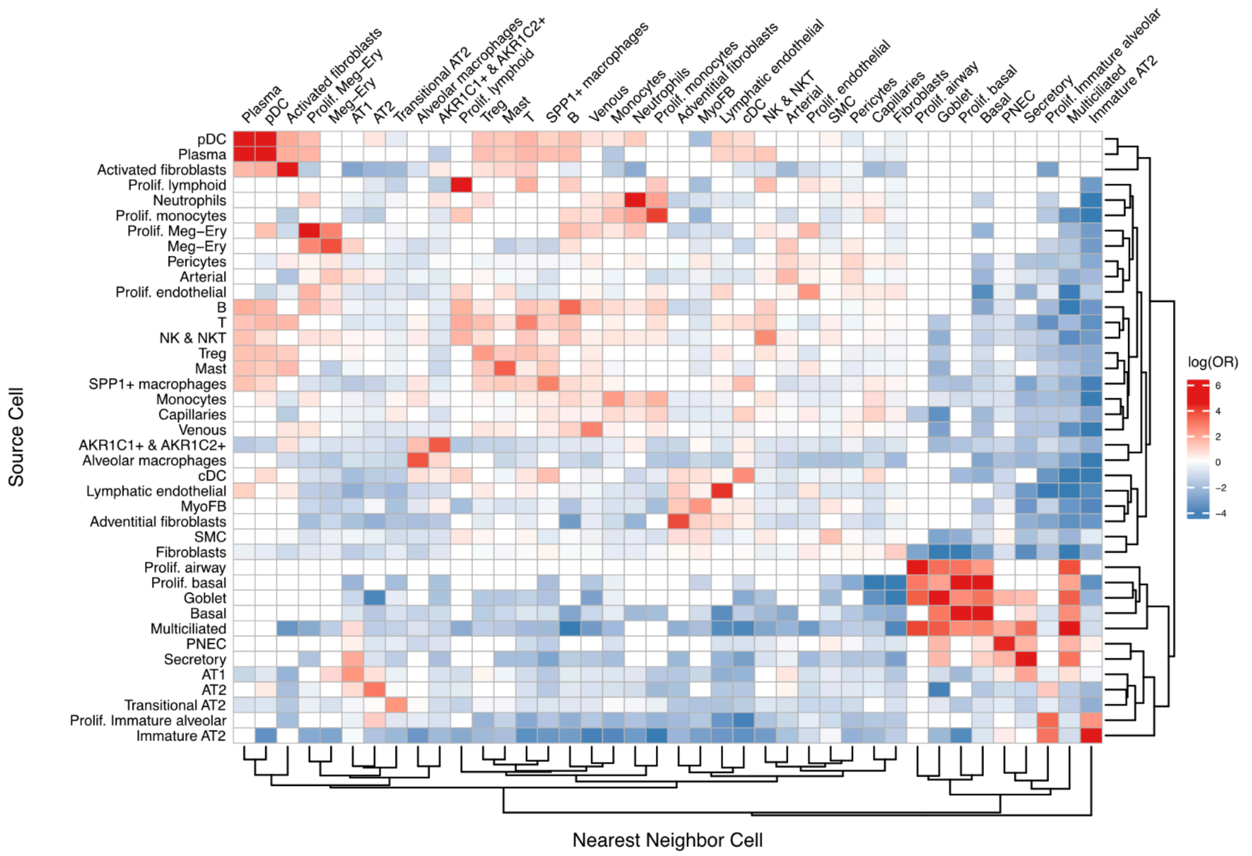

Supplemental Fig. S6:

Cell proximity results across all samples. This heatmap shows the log odds ratio (log(OR)) for each cell type pair, calculated with a Fisher's Exact test (**see Methods**). Positive values (red) indicate that the cells in that cell type pair are more likely to be located close to one another, while negative values (blue) indicate that those cells are less likely to be located close to each other. Non-significant results (FDR > 0.1) are coded as 0. See Supplemental figures 7-14 for cell proximity results across categories and specific samples representing rare diseases.

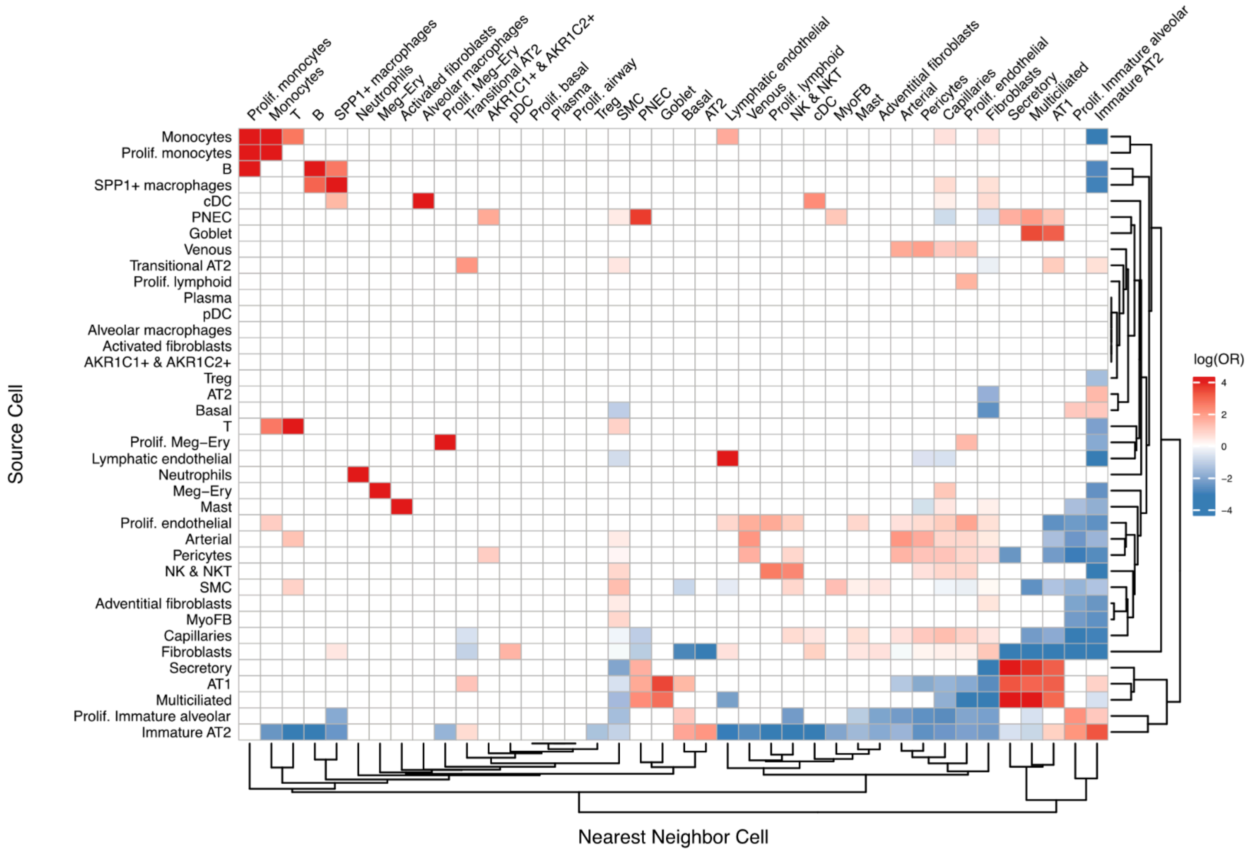

Supplemental Fig. S7:

Cell proximity results across all samples grouped into “early canalicular”.

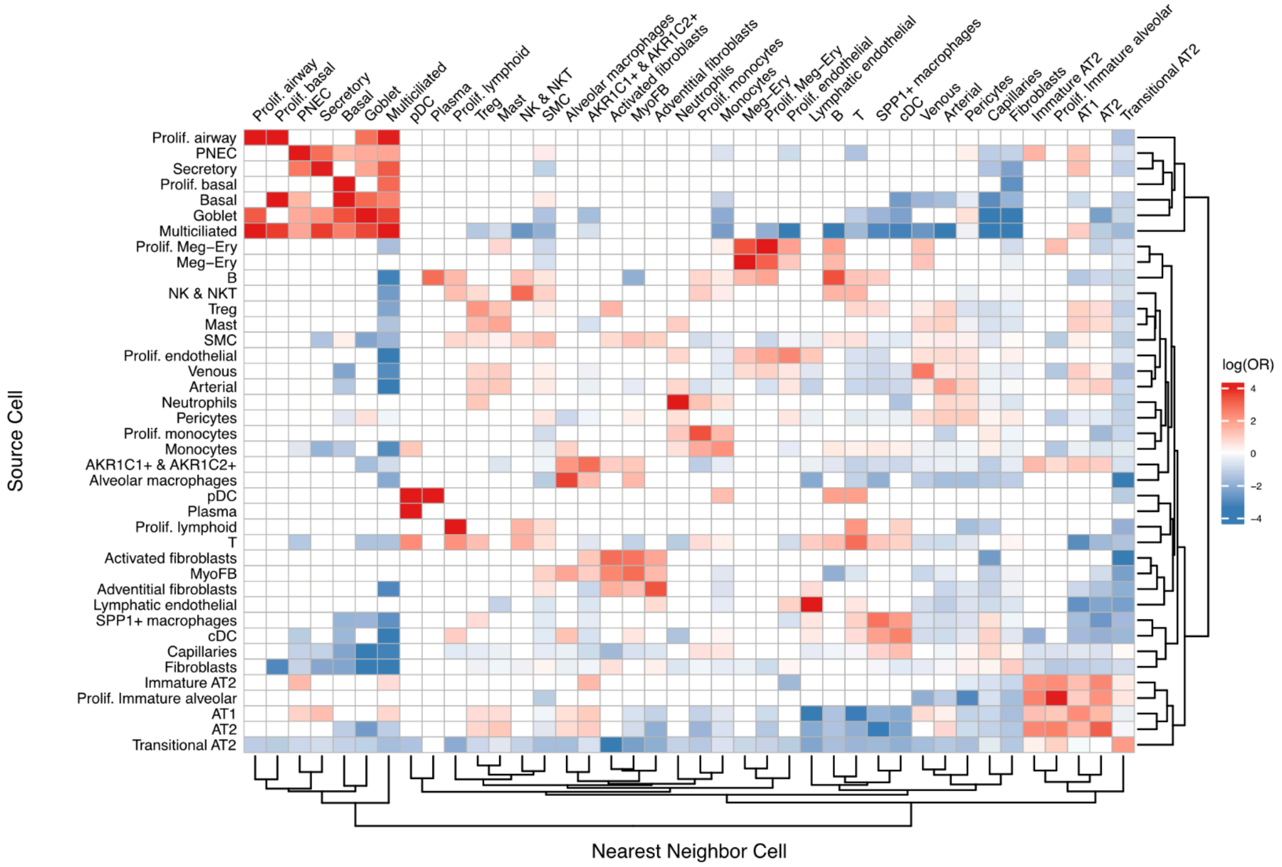

Supplemental Fig. S8:

Cell proximity results across all samples grouped into “late canalicular”.

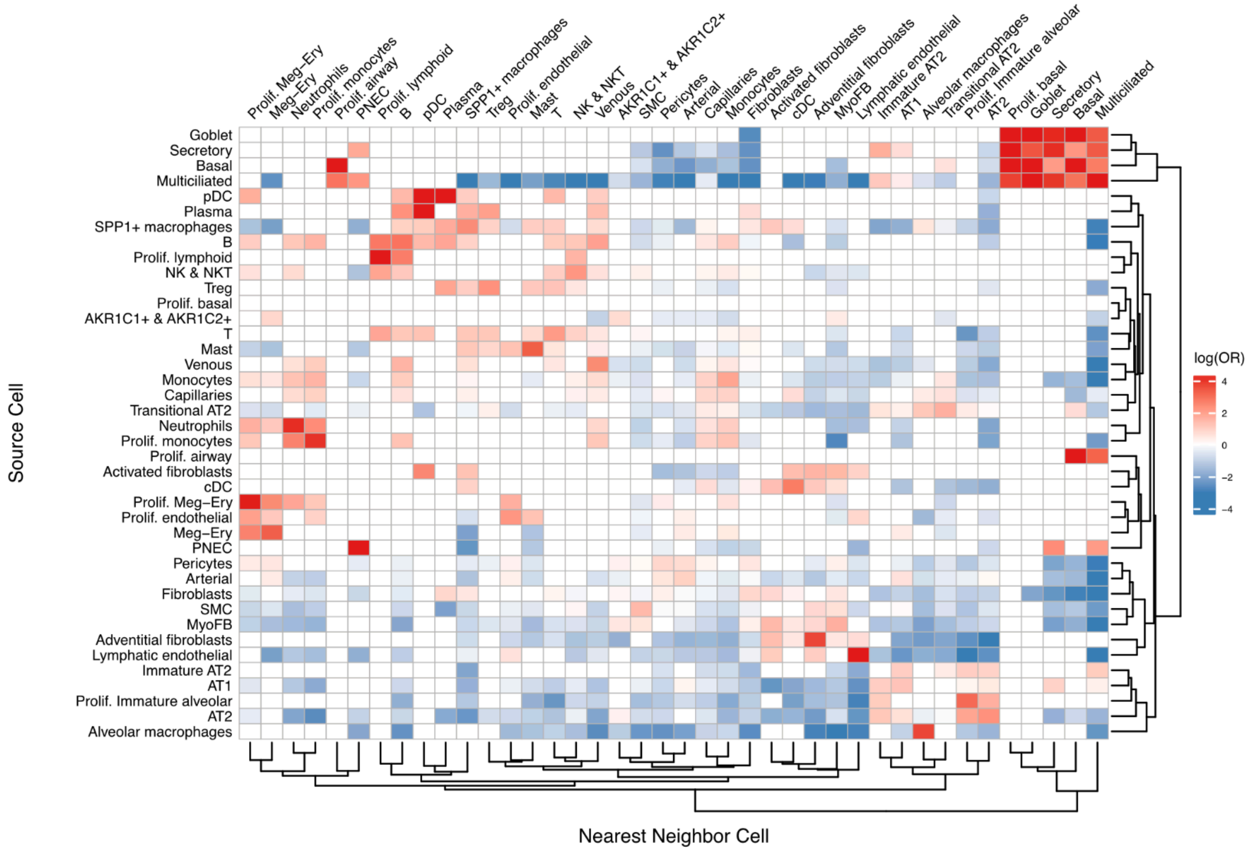

Supplemental Fig. S9:

Cell proximity results across all samples grouped into “saccular”.

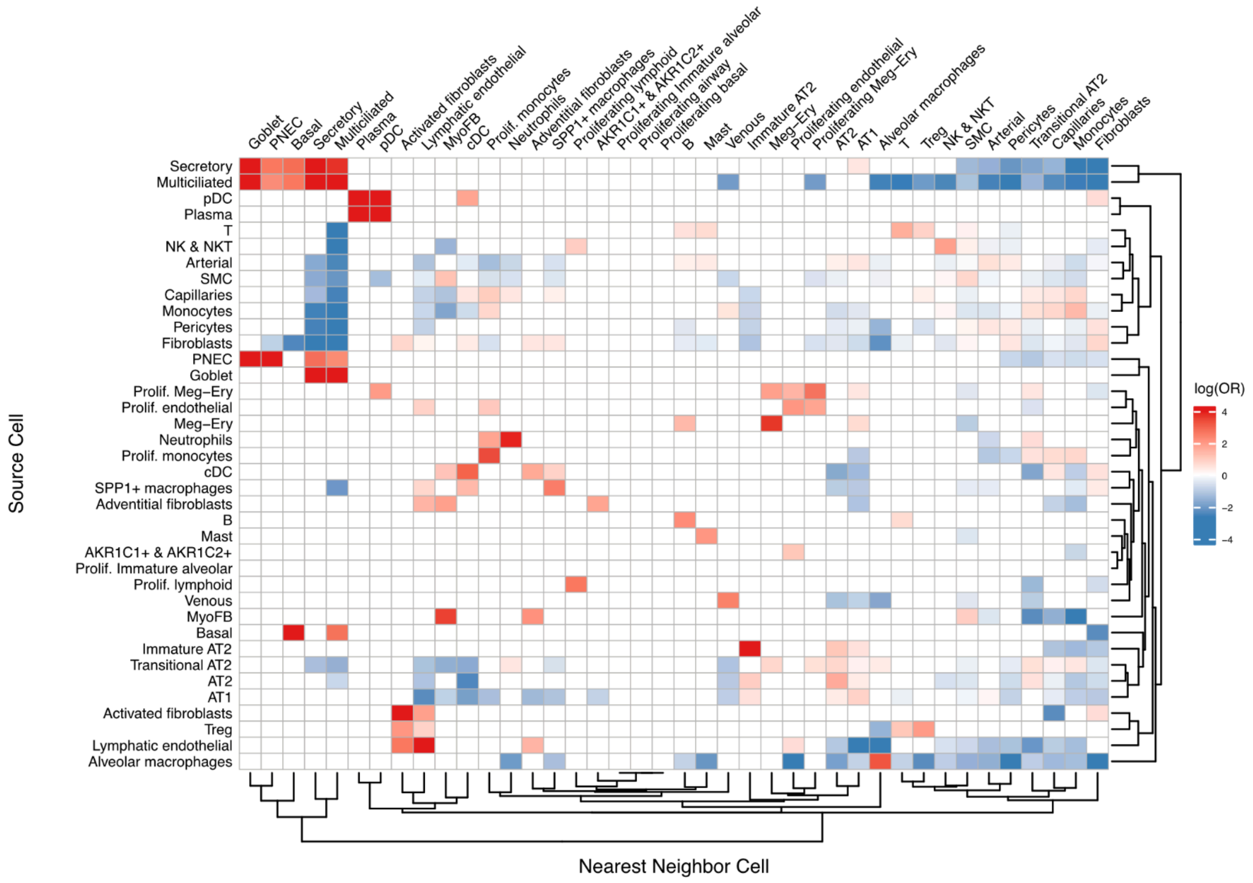

Supplemental Fig. S10:

Cell proximity results across all samples grouped into “alveolar”.

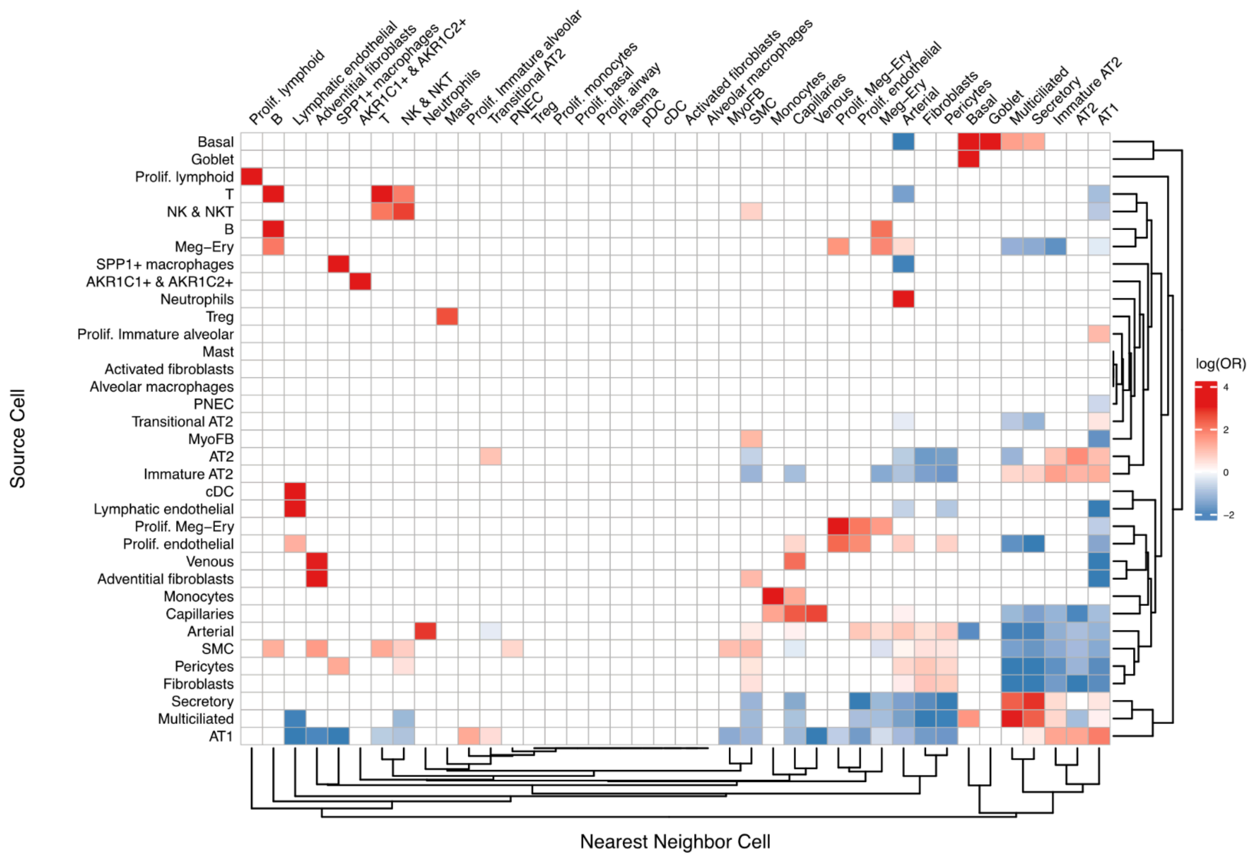

Supplemental Fig. S11:

Cell proximity results across one individual (PDL014) diagnosed with CHAOS syndrome who fell within the “rare disease” grouping.

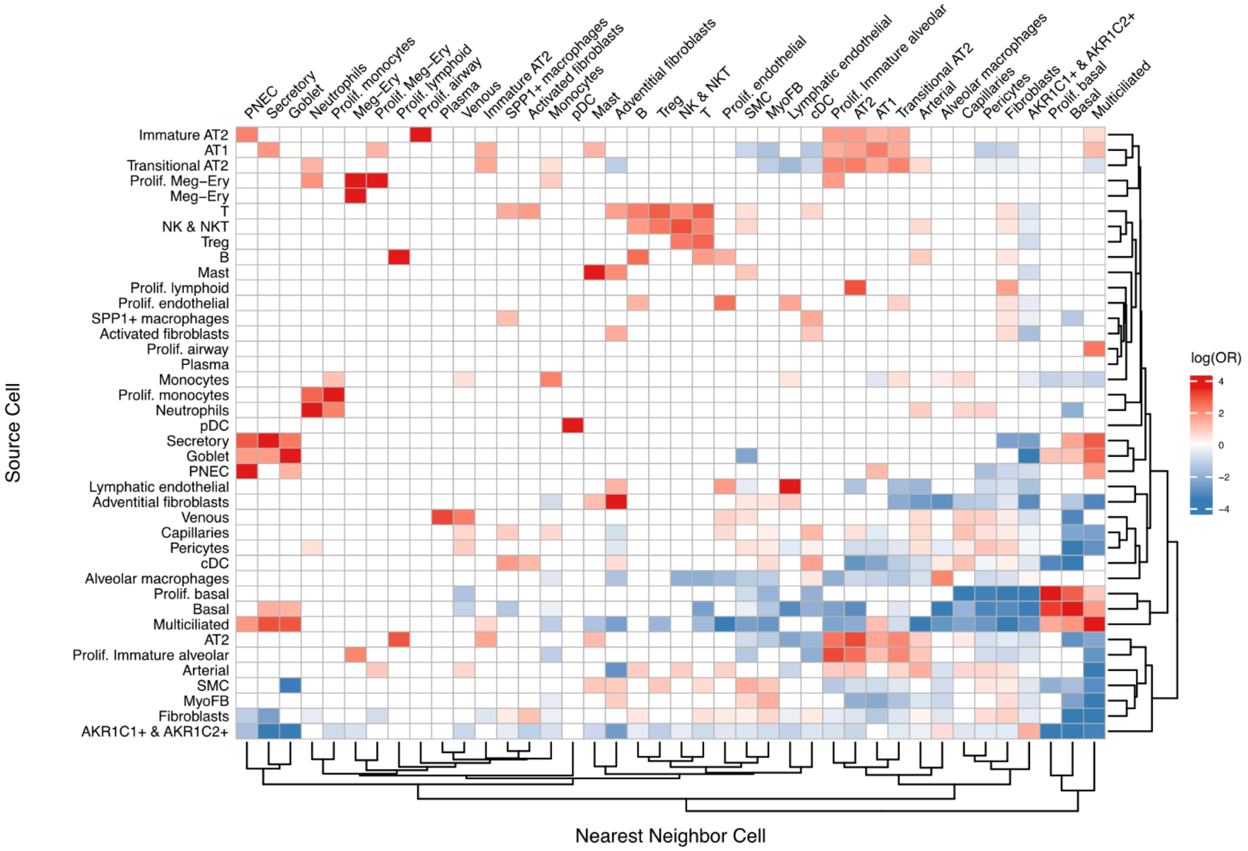

Supplemental Fig. S12:

Cell proximity results across one individual (PDL015) diagnosed with pulmonary hypoplasia and dysplastic kidneys who fell within the “rare disease” grouping.

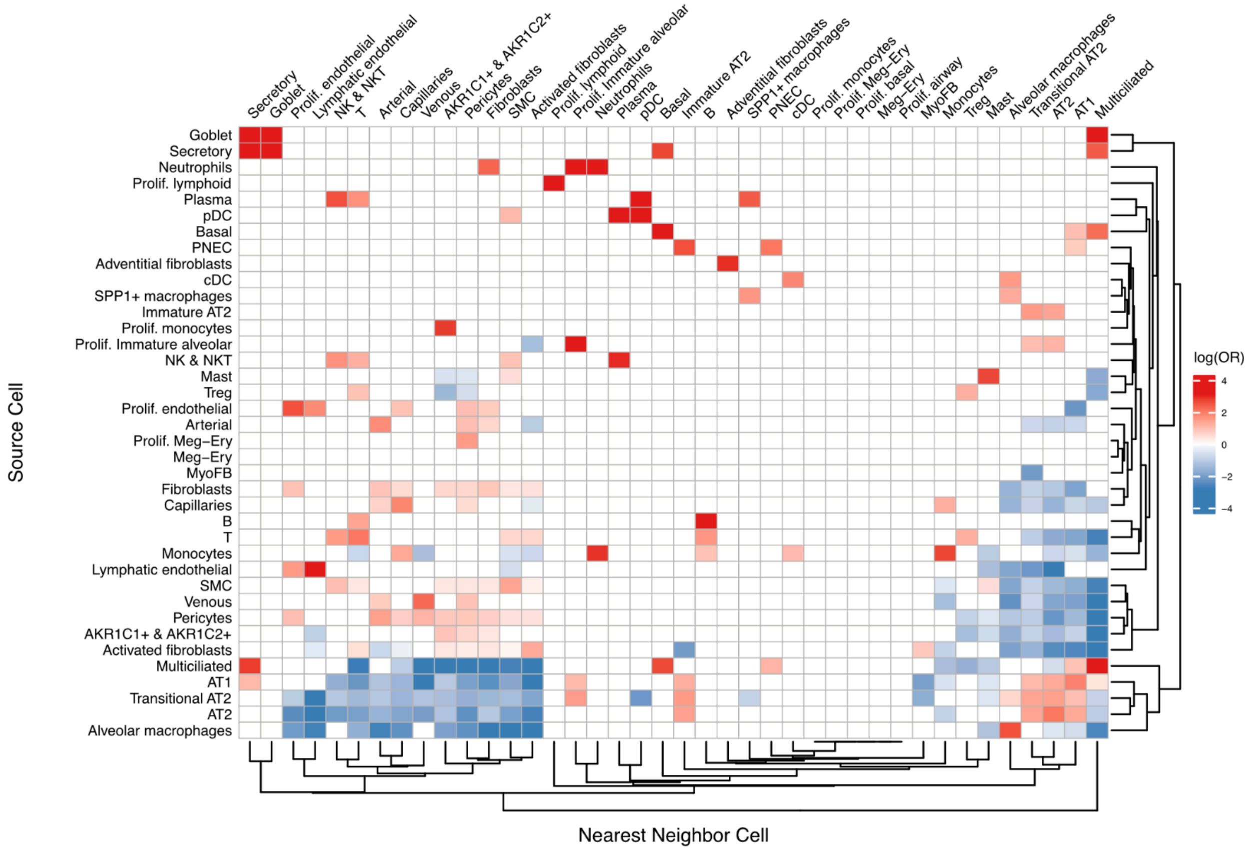

Supplemental Fig. S13:

Cell proximity results across one individual (PDL016) diagnosed with ARB fetopathy and severe BPD who fell within the “rare disease” grouping.

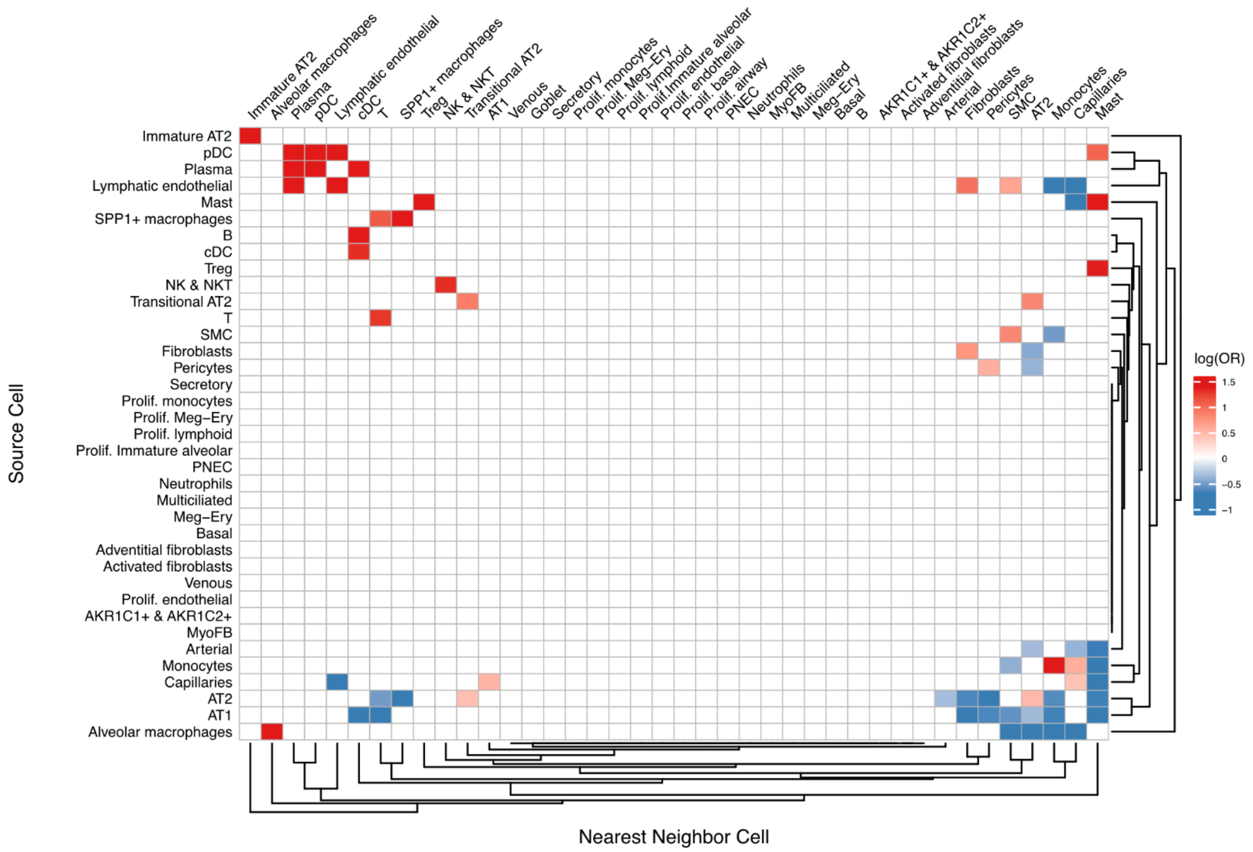

Supplemental Fig. S14:

Cell proximity results across one sample (PDL017) that was an explant of an adult aged 30 years living with BPD who fell within the “rare disease” grouping.

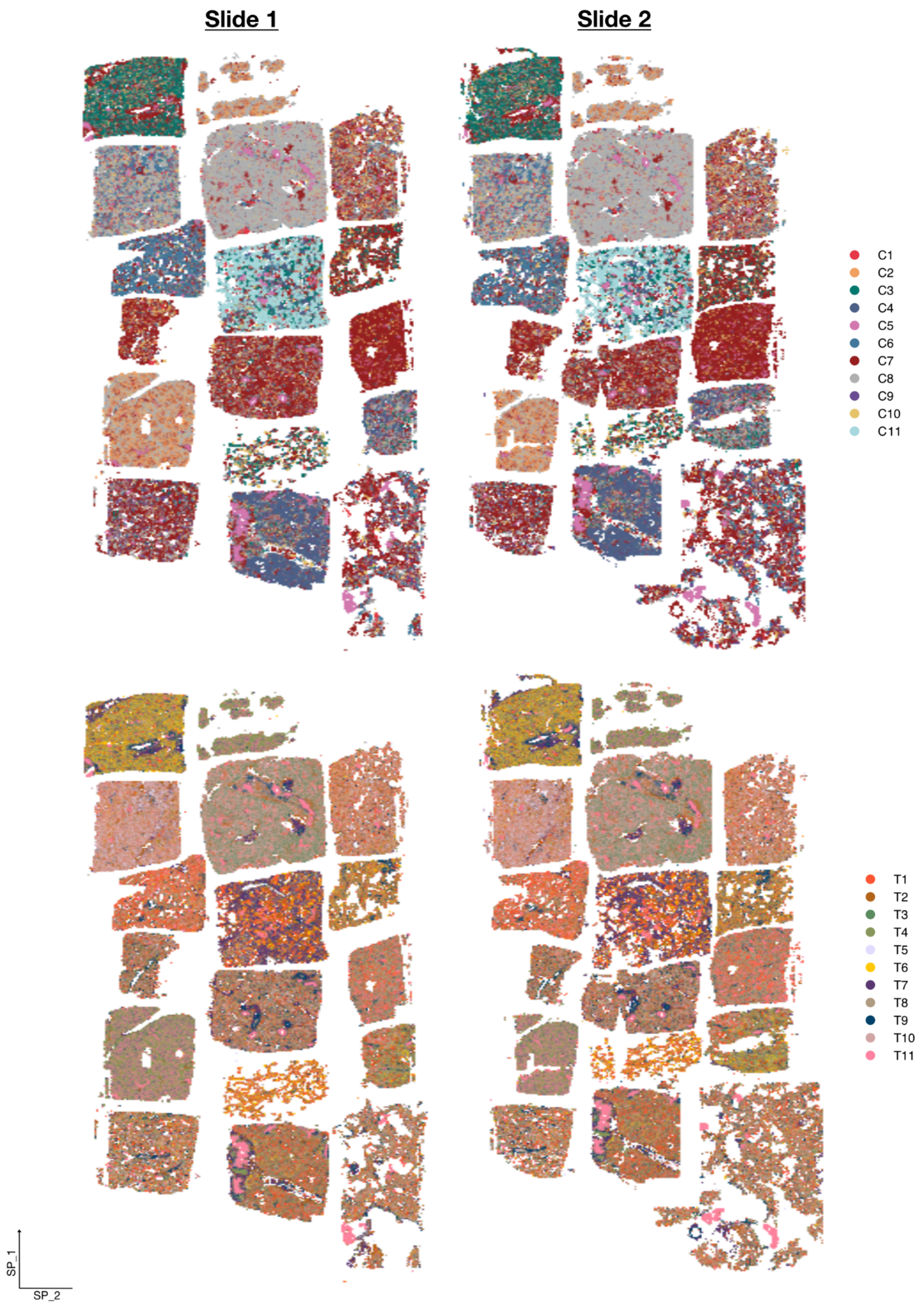

Supplemental Fig. S15:

Plots that show the structure of each tissue sample and showcase the distribution of niches across each sample, split across both slides. The top images are colored by the cell niches, and the bottom are colored by the transcript niches.

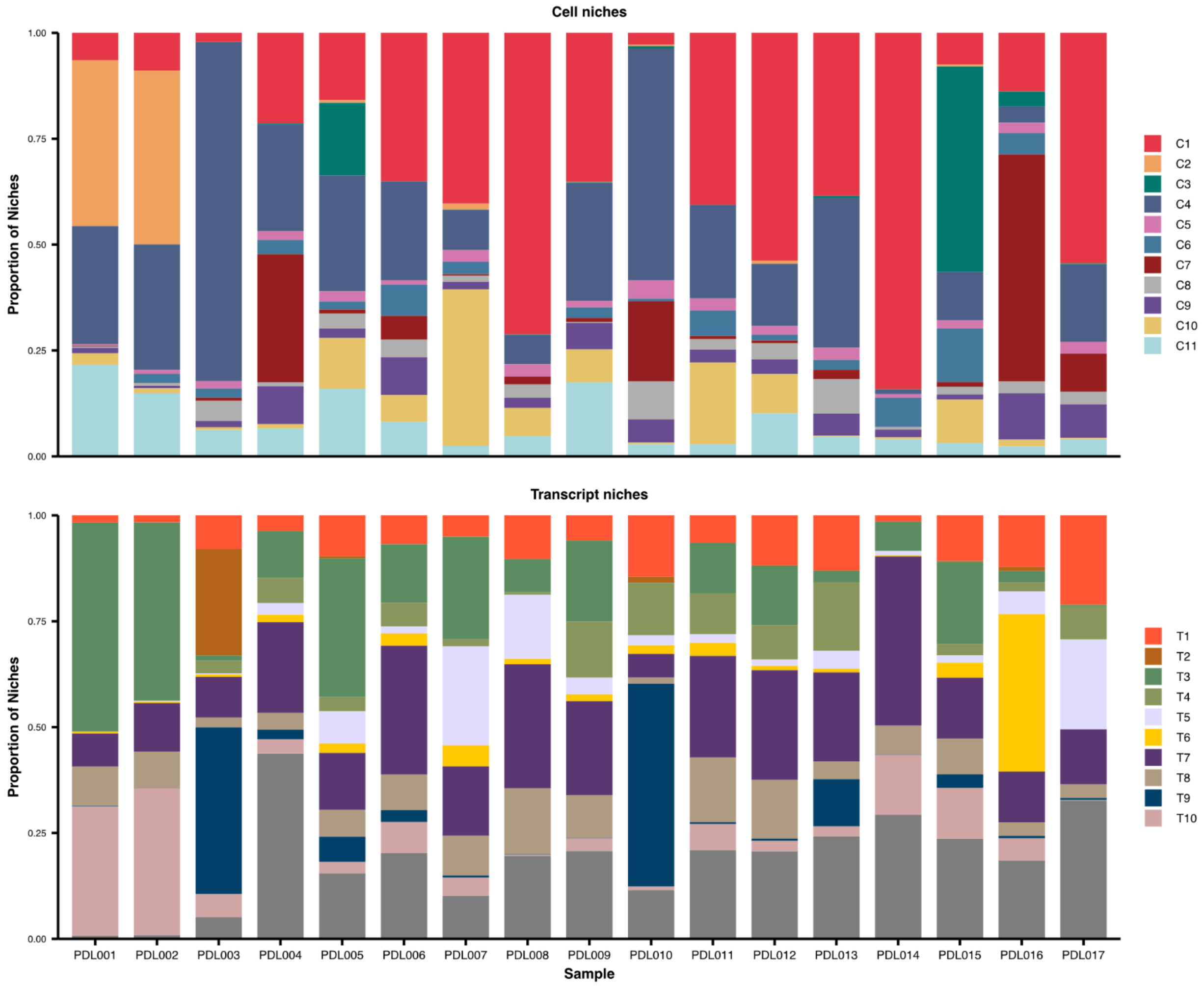

Supplemental Fig. S16:

Stacked bar plots show the composition of cell niches (top) and transcript niches (bottom) split across each individual.

a.

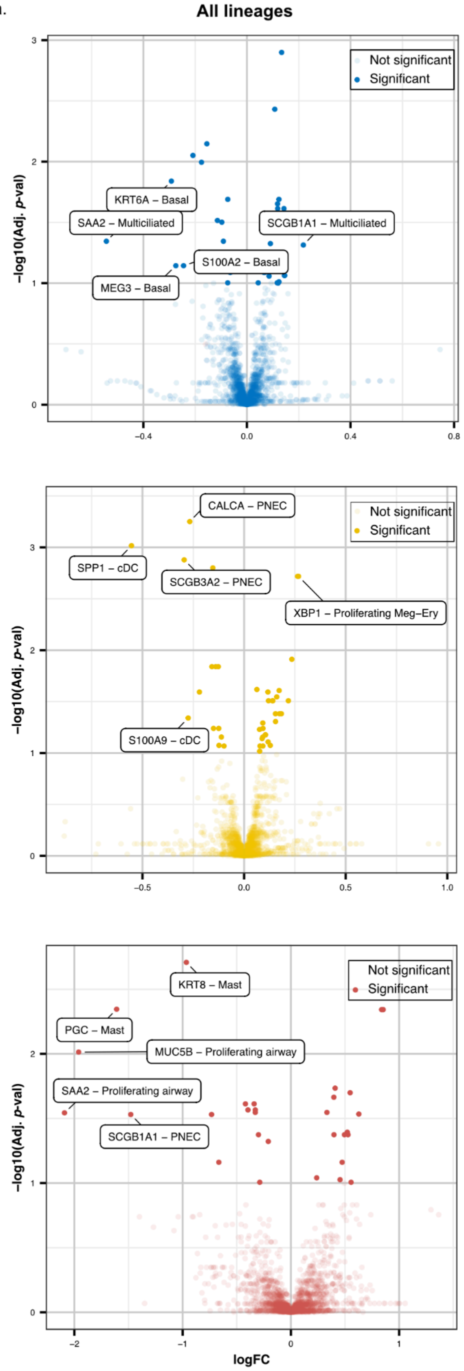

b.

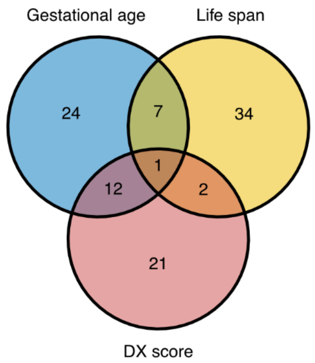

Gestational age
AGR3 - Basal
COL1A1 - Proliferating lymphoid
EPAS1 - T
FCGR3A - Proliferating lymphoid
GZMA - Proliferating lymphoid
HERPUD1 - *SPP1*+ macrophages
HIST1H1C - *SPP1*+ macrophages
HMGA1 - *SPP1*+ macrophages
ICAM1 - Basal
KRT6A - Basal
LYZ - *SPP1*+ macrophages
MEG3 - Basal
MEG3 - Proliferating Meg-Ery
NKG7 - Proliferating lymphoid
PDGFRA - Fibroblasts
PDGFRA - SPP1+ macrophages
PDGFRA - T
PECAM1 - T
RNASE1 - Basal
SAA2 - Multiciliated
SCGB1A1 - Multiciliated
SEC11C - Multiciliated
SFTPD - AT2
SOX2 - Multiciliated

DX score
HERPUD1 - AT2
PGC - Mast
KRT8 - Mast
CTNNB1 - Mast
HIF1A - Mast
ATF4 - Mast
ITGB1 - Mast
ACTA2 - Mast
SPARCL1 - Mast
HYOU1 - Mast
RHOA - Mast
HERPUD1 - Multiciliated
COL1A1 - Plasma
LUM - Plasma
COL3A1 - Plasma
HERPUD1 - Plasma
COL1A2 - Plasma
SCGB1A1 - PNEC
EPCAM - PNEC
HIF1A - PNEC
SAA2 - Proliferating airway
MUC5B - Proliferating airway
MEG3 - *SPP1*+ macrophages
ICAM1 - *SPP1*+ macrophages

Life span
ACTA2 - Meg-Ery
ATG7 - cDC
ATP2A3 - Meg-Ery
COL1A1 - Secretory
COL1A2 - Proliferating Meg-Ery
CTNNB1 - Meg-Ery
CTNNB1 - PNEC
CTNNB1 - Secretory
EHMT1 - Secretory
EPAS1 - Secretory
FN1 - Meg-Ery
HERPUD1 - Proliferating Meg-Ery
HIST1H1C - Proliferating Meg-Ery
HSPA5 - Meg-Ery
ICAM1 - Secretory
ITGB1 - PNEC
MGST1 - PNEC
PDIA4 - Proliferating Meg-Ery
PGC - Secretory
PKM - PNEC
RHOA - PNEC
S100A9 - cDC
SCGB3A2 - PNEC
SEC11C - Secretory
SNCA - Meg-Ery
SPARCL1 - Secretory
SPP1 - cDC
STAT6 - Secretory
UBE2J1 - Proliferating Meg-Ery
VIM - PNEC
WWTR1 - Secretory
XBP1 - PNEC
XBP1 - Proliferating Meg-Ery
YAP1 - PNEC

Gestational age and life span
CALCA - PNEC
CPA3 - Mast
EPAS1 - PNEC
HIF1A - Multiciliated
IL7R - Arterial
LYZ - Alveolar macrophages
MEG3 - Arterial

Gestational age and DX score
CCL2 - Mast
HMGA1 - Multiciliated
ICAM1 - Mast
ICAM1 - *SPP1*+ macrophages
KIT - Mast
LUM - Mast
MEG3 - Mast
MEG3 - *SPP1*+ macrophages
PDGFRA - Mast
S100A2 - Basal
VEGFA - Mast
YAP1 - Mast

Life span and DX score
COL3A1 - Plasma
SEC11C - PNEC

All three terms
TPSAB1 - Mast

Supplemental Fig. S17:

a) Volcano plots showing the top 5 gene-cell type pairs in all lineages with the highest logFC per gestational age (blue), life span (yellow), and disease severity score (red). LogFC values in both were generated by a linear model we built that utilized the Limma package in R (**see Methods**). b) A venn diagram showing the overlapping gene-cell type pairs across all three terms within all lineages.

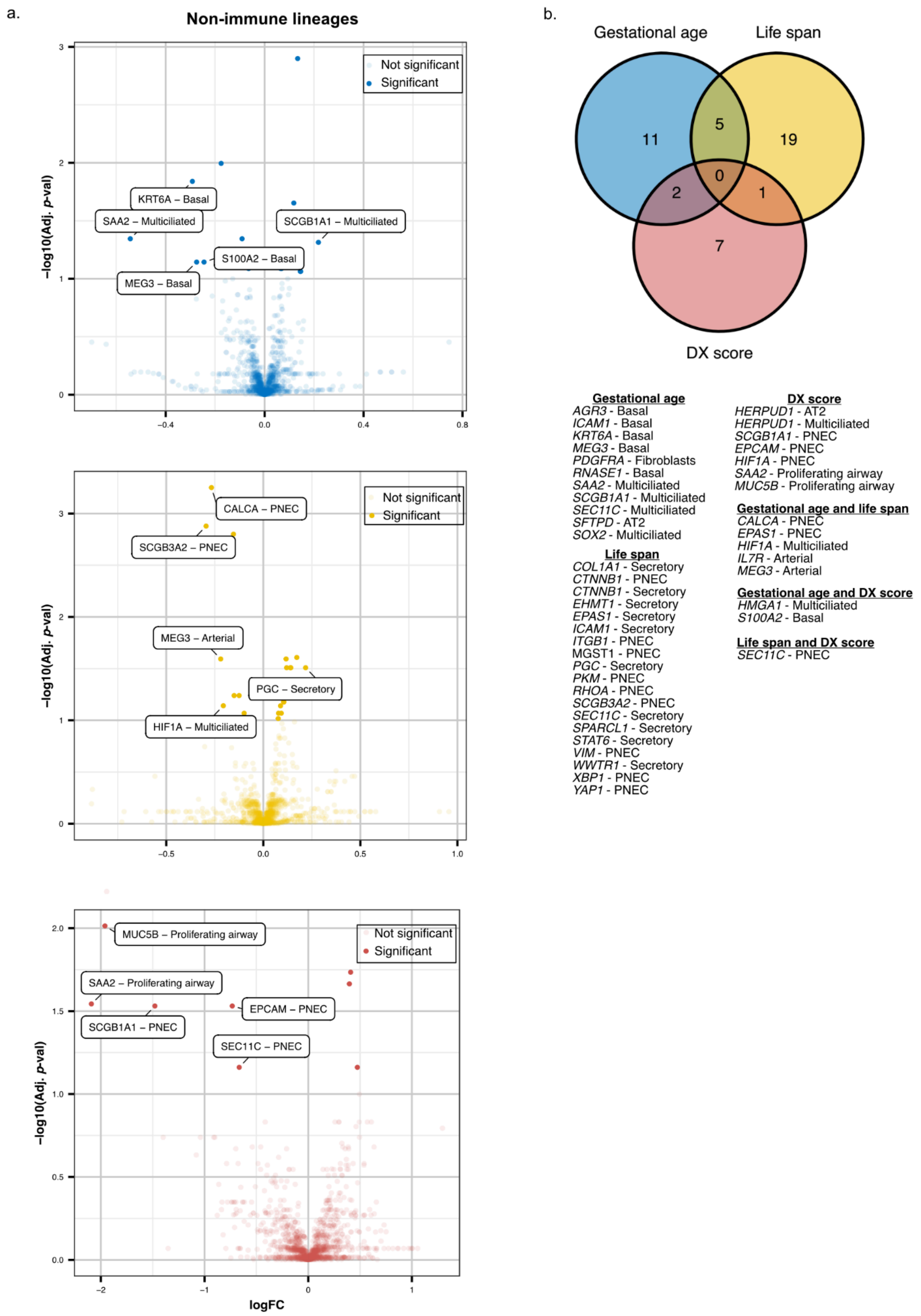

Supplemental Fig. S18:

a) Volcano plots showing the top 5 gene-cell type pairs in epithelial, endothelial, and mesenchymal lineages with the highest logFC per gestational age (blue), life span (yellow), and disease severity score (red). LogFC values in both were generated by a linear model we built that utilized the Limma package in R (**see Methods**). b) A venn diagram showing the overlapping gene-cell type pairs across all three terms within the epithelial, endothelial, and mesenchymal lineages.
